# Female reproductive factors and the risk of lung cancer in postmenopausal women: a nationwide cohort study

**DOI:** 10.1038/s41416-020-0789-7

**Published:** 2020-03-17

**Authors:** Keun Hye Jeon, Dong Wook Shin, Kyungdo Han, Dahye Kim, Jung Eun Yoo, Su-Min Jeong, Jong ho Cho

**Affiliations:** 1 Department of Family Medicine and Supportive Care Centre, Samsung Medical Centre, Sungkyunkwan University School of Medicine, Seoul, Korea; 2 Department of Digital Health, Samsung Advanced Institute for Health Science and Technology (SAIHST), Sungkyunkwan University, Seoul, Korea; 3 Department of Medical Statistics, The Catholic University of Korea, Seoul, Korea; 4 Department of Economics and Centre for Economic and Social Research, University of Southern California, Los Angeles, and RAND Corporation, Santa Monica, CA USA; 5 Department of Nutrition Epidemiology, TH Chan School of Public Health, Harvard University, Boston, MA USA; 6 Department of Thoracic Surgery, Samsung Medical Centre, Sungkyunkwan University School of Medicine, Seoul, Korea

**Keywords:** Cancer epidemiology, Lung cancer

## Abstract

**Background:**

Reproductive factors and hormone use in postmenopausal women have been hypothesised to affect the risk of developing lung cancer, but the epidemiological evidence is inconsistent.

**Methods:**

Using the Korean National Health Insurance System database, we identified 4,775,398 postmenopausal women older than 40 years who had undergone both cardiovascular health- and cancer screening between 1 January 2009 and 31 December 2014. Information about reproductive factors was obtained from a self-administered questionnaire. The risk of lung cancer was estimated using Cox proportional hazard regression models.

**Results:**

During a median follow-up of 4.4 years, 16,556 women (15,223 non-smokers) were diagnosed with lung cancer. The risk of lung cancer was not significantly influenced by early menarche age (adjusted hazard ratio [aHR] 1.03 for menarche ≥18 vs. ≤14; 95% confidence interval [CI], 0.98–1.09) or late age at menopause (aHR 1.02 for menopause ≥55 vs. <40; 95% CI, 0.91–1.14). Furthermore, the number of children, duration of breastfeeding and use of hormone replacement therapy were not associated with the risk of lung cancer.

**Conclusions:**

No statistically significant association was found between reproductive factors and the risk of lung cancer in postmenopausal Korean women.

## Background

Lung cancer is the leading cause of cancer deaths worldwide, and the third most common cancer in women, after breast- and colorectal cancer.^1^ In most countries, including Korea, the incidence of lung cancer in women is increasing, whereas in men, it has decreased or stabilised.^2,3^

Lung cancer in women has several characteristics that differ from lung cancer in men. Although smoking is the most important risk factor, a significant portion of lung cancer in women occurs in non-smokers.^4^ Global statistics estimate that 20% of lung cancer in Western populations develops in non-smokers.^1^ In Korea, 38% of female non-small-cell lung cancer patients reported themselves to be non-smokers.^5^ Female lung cancer patients have a higher proportion of adenocarcinoma histology,^6^ and a higher frequency of mutations in the epidermal growth factor receptor (EGFR)^7^ than male lung cancer patients. Such sex-related differences suggest that hormonal factors might influence the development of lung cancer.

Hormonal involvement in lung carcinogenesis was first suggested by experimental studies that identified oestrogen and progesterone receptors in human lung cancers.^8^ Steroid hormone-related receptors, including oestrogen receptors, progesterone receptors and human epidermal growth factor receptor 2, are frequently expressed in tumour lung tissue,^8–10^ and have been linked to survival in lung cancer patients.^11,12^ Oestrogen receptor α nuclear expression correlates significantly with adenocarcinoma histology, female sex and a history of never smoking.^9^ In addition, a recent study revealed a sex-related difference in oestrogen receptor β nuclear expression.^8^

As a result, it has been hypothesised that reproductive factors and hormone use could affect women’s risk of developing lung cancer. However, the epidemiological evidence is inconsistent and does not fully support that hypothesis. For example, with respect to late age at menopause, studies have variously shown an increase,^13,14^ a decrease^15,16^ or no significant change^17–19^ in lung cancer risk; early age at menarche has been shown to increase^15^ or have no significant effect^16,18–20^ on risk; a small number of pregnancies has been shown to increase,^19,21,22^ decrease^20^ and have no significant effect^15,18^ on risk; late age at first birth might decrease^20^ or have no significant effect^15,16,18,22^ on risk. Studies on the use of hormones have also produced heterogeneous results: the use of oral contraceptives has been shown to increase^16^ or have no significant effect^18–20,22–26^ on risk, and the use of hormone replacement therapy (HRT) might increase,^27,28^ decrease^17,29,30^ or have no appreciable influence^15,18,19,25,31,32^ on risk.

Previous cohort studies have been limited by small sample size (cohort studies published between 2001 and 2015 included 87–3512 lung cancer cases^15–22,27,29–34^), and case–control designs prone to recall bias. Moreover, the potential association between hormone exposure and lung cancer has not been comprehensively elucidated because several studies focused exclusively on exogenous hormone use.^25,27–32,35^

Furthermore, even though lung cancer in East Asia shows unique patterns in epidemiology^7^ and treatment response,^36^ few studies have been conducted there. East Asian females with lung cancer generally show lower smoking prevalence (9.9% in Korea,^5^ 5.2% in China^7^ and 17–25.6% in Japan^37^) than is common in Western countries. Several case–control studies have been carried out in China and Singapore, but only a few cohort studies have been conducted in Asian countries—1 in Japan,^18^ 2 in China^22,33^ and 2 in Singapore.^19,21^

In this study, we investigate the association between reproductive factors and the risk of lung cancer among postmenopausal women in a nationwide population cohort in Korea.

## Methods

### Data source and study setting

The Korean National Health Insurance Service (NHIS) is the single insurer that provides universal insurance for ~97% of the South Korean population. The remaining 3% of the population (in the lowest-income bracket) is covered by the Medical Aid program financed by the government, but it is also administered by the NHIS.

The NHIS provides national health check-up programmes, including a general health examination focused on cardiovascular risk factors, for all citizens aged 40 and above, and all employees regardless of age.^38^ It records the results of those general health examinations and questionnaires on lifestyle behaviour. In addition, the NHIS provides the National Cancer Screening Program (NCSP),^39^ which screens all individuals for stomach, liver, colorectal, breast and cervical cancers, as indicated by age.^39^ During our study period, Korean women were eligible to participate in biennial screening for breast cancer beginning at age 40 and cervical cancer beginning at age 30.^39^

The NHIS has qualification data on all its enrolees (age, sex, income level and mortality), diagnosis codes from the International Classification of Disease 10th revision (ICD-10), medical treatment information (medical services provided for medical expense claims) and prescription information.^40,41^ To manage enrolee qualification, the NHIS updates its mortality data through frequent links with mortality data from the Korean National Statistical Office. The NHIS database is widely used in epidemiological studies investigating disease incidence,^42–44^ and the details of the database profiles are described elsewhere.^38,40,45,46^ This study was approved by the Institutional Review Board of Samsung Medical Centre (IRB File No. SMC 2018-05-020).

### Study population and data collection

**Fig. 1:**
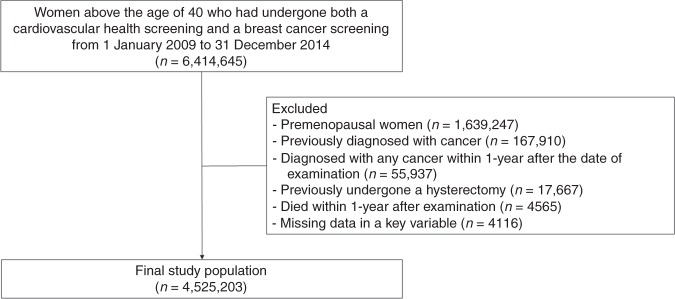
Flow chart of the cohort study design.

From the NHIS database, we collected data for women older than 40 who had undergone both a cardiovascular health screening and an NCSP (breast/cervical cancer) screening between 1 January 2009 and 31 December 2014. Among 4,775,398 postmenopausal women who qualified, those who had previously been diagnosed with cancer (*n* = 167,910), participants diagnosed with any cancer within 1 year after the date of examination (*n* = 55,937), individuals who had previously undergone a hysterectomy (*n* = 17,667), participants who died within 1 year after examination (*n* = 4565) and those missing data in a key variable (*n* = 4116) were excluded. We excluded those who had previously undergone a hysterectomy because the questionnaire provided no information about the timing of the hysterectomy, and whether a bilateral oophorectomy had been performed concurrently. After those exclusions, a total of 4,525,203 individuals were included in our analyses (Fig. 1).

Information about health-related behaviours, and the menstrual and reproductive histories of participants, was obtained using a self-administered questionnaire. Participants came to the medical centre on the day of the screening examination, and filled out the questionnaire prior to the exam. According to the NCSP guidelines, women were asked about their age at menarche and age at menopause as continuous variables. For analysis purposes, we categorised age at menarche as ≤14 years, 15–16 years, 17 years and ≥18 years, and age at menopause as <40 years, 40–44 years, 45–49 years, 50–54 years and ≥55 years. The reproductive period was calculated as the interval between the age at menarche and age at menopause. Information on parity number, total lifetime breastfeeding history, HRT history and the use of oral contraceptives was collected categorically: parity number (0, 1 or ≥2 children), total lifetime breastfeeding history (never, <6 months, 6–12 months and ≥12 total months), duration of HRT (never, <2 years, 2–5 years, ≥5 years and unknown) and duration of oral contraceptive use (never, <1 year, ≥1 year and unknown).

Participants’ history of smoking was classified as never smoked, former and <10 pack-years smoker, former and ≥10 pack-years smoker, current and <10 pack-years smoker and current and ≥10 pack-years smoker. Drinking was divided into three levels: none, mild (<30 g of alcohol/day) and heavy (≥30 g/day). Regular exercise was defined as moderate physical activity for more than 30 min, and more than 5 days per week during the past week. Body mass index (BMI) was calculated using weight (kg) divided by height in metres squared (m^2^), and classified as low (<18.8 kg/m^2^), normal to overweight (18.5–24.9 kg/m^2^) and obese (≥25 kg/m^2^). Hypertension, diabetes and dyslipidaemia were defined using physicians’ diagnoses or the use of medication, based on self-reporting. Household income was categorised into quartiles based on insurance premium levels (in Korea, insurance premiums are determined by income level), with those covered by Medical Aid (poorest 3%) being merged into the lowest-income quartile.

### Study outcomes and follow-up

The primary endpoint of this study was newly diagnosed lung cancer, defined as new claims for inpatient or outpatient care with the ICD-10 diagnosis code of C34 (malignant neoplasm of lung and bronchus), and registration in the special co-payment reduction programme for critical illnesses. The NHIS special co-payment reduction programme for cancer and some other intractable diseases relieves the large financial burden of patients with serious and rare diseases. For example, cancer patients pay only 5% of the total medical bill incurred for cancer diagnostic tests or treatment. As enrolment in this programme requires a medical certificate from a physician, the cancer diagnosis variable in our study is considered sufficiently reliable, and has been used in other studies.^42,44^ The cohort was followed from 1 year after the health check-up date to the date of incident lung cancer, death or the end of the study period (31 December 2016), whichever came first.

### Statistical analysis

Continuous variables are presented as the mean ± standard deviation, and categorical variables are presented as the number and percentage. The incidence rates of lung cancer were calculated by dividing the number of incident cases by the total follow-up period. Hazard ratios (HRs) and 95% confidence interval (CI) values for lung cancer were analysed using Cox proportional hazard models for various reproductive factors. A multivariable-adjusted proportional hazard model was applied: (1) model 1 was age-adjusted; (2) model 2 was adjusted for age at menarche and menopause, parity, duration of breastfeeding, duration of HRT, duration of oral contraceptive use, alcohol consumption, smoking, regular exercise, income, BMI, hypertension, diabetes mellitus, dyslipidaemia and cancer; (3) model 3 was adjusted for reproductive period instead of age at menarche and menopause separately, as in model 2. Statistical analyses were performed using SAS version 9.4 (SAS Institute Inc., Cary, NC, USA), and a *P* value <0.05 was considered statistically significant.

## Results

### Baseline characteristics of study participants

During a mean follow-up of 4.4 years (19,910,975 person-years), 16,556 women (15,223 non-smokers) were diagnosed with lung cancer, for an incidence rate of 83.15 cases per 100,000 person-years. Table 1 provides the baseline information and characteristics of the study population. The mean age of the study participants was 61.2 years. Among all participants, only 3.1% were current smokers, 91.9% had breastfed and 14.6% had received HRT.

**Table 1:** Selected baseline characteristics of the study population.

Variable	Total (*N* = 4,525,203)	Women not diagnosed with lung cancer (*N* = 4,508,647)	Women diagnosed with lung cancer (*N* = 16,556)
Mean age, y (SD)	61.2 (8.7)	61.3 (8.7)	65.9 (8.7)
*Age at menarche, no. (%)*			
Mean (SD)	16.3 (1.9)	16.3 (1.9)	16.6 (1.9)
≤14 y	712,179 (15.7)	710,254 (15.8)	1925 (11.6)
15–16 y	1,806,866 (39.9)	1,800,693 (39.9)	6173 (37.3)
17 y	854,123 (18.9)	850,758 (18.9)	3365 (20.3)
≥18 y	1,152,035 (25.5)	1,146,942 (25.4)	5093 (30.8)
*Age at menopause, no. (%)*			
Mean (SD)	50.2 (4.0)	50.2 (4.0)	50.0 (4.4)
<40 y	73,474 (1.6)	73,120 (1.6)	354 (2.1)
40–44 y	237,584 (5.3)	236,500 (5.3)	1084 (6.6)
45–49 y	1,172,184 (26.0)	1,167,918 (25.9)	4266 (25.8)
50–54 y	2,510,733 (55.5)	2,501,988 (55.5)	8745 (52.8)
≥55 y	531,228 (11.7)	529,121 (11.7)	2107 (12.7)
*Reproductive period, no. (%)*			
Mean (SD)	33.9 (4.4)	33.9 (4.4)	33.4 (4.7)
<30 y	562,807 (12.4)	560,192 (12.4)	2615 (15.8)
30–34 y	1,768,024 (39.1)	1,761,249 (39.1)	6775 (40.9)
35–39 y	1,844,270 (40.8)	1,838,391 (40.8)	5879 (35.5)
≥40	350,102 (7.7)	348,815 (7.7)	1287 (7.8)
*Parity, no. (%)*			
Nulliparous	99,224 (2.2)	98,847 (2.2)	377 (2.3)
1 child	347,461 (7.7)	346,491 (7.7)	970 (5.9)
≥2 children	4,078,518 (90.1)	4,063,309 (90.1)	15,209 (91.9)
*Duration of breastfeeding, no. (%)*			
Never	366,238 (8.1)	365,176 (8.1)	1062 (6.4)
<0.5 y	362,575 (8.0)	361,738 (8.0)	837 (5.1)
0.5 to < 1 y	804,665 (17.8)	802,226 (17.8)	2439 (14.7)
≥1 y	2,991,725 (66.1)	2,979,507 (66.1)	12,218 (73.8)
*Hormone therapy, no. (%)*			
Never used	3,696,024 (81.7)	3,682,200 (81.7)	13,824 (83.5)
<2 y	392,482 (8.7)	391,279 (8.7)	1203 (7.3)
2 to <5 y	150,713 (3.3)	150,263 (3.3)	450 (2.7)
≥5 y	119,890 (2.7)	119,457 (2.7)	433 (2.6)
Missing	166,094 (3.7)	165,448 (3.7)	646 (3.9)
*Oral contraceptive use, no. (%)*			
Never used	3,646,833 (80.6)	3,633,551 (80.6)	13,282 (80.2)
<1 y	397,111 (8.8)	395,766 (8.8)	1345 (8.1)
≥1 y	261,279 (5.8)	260,236 (5.8)	1043 (6.3)
Missing	219,980 (4.9)	219,094 (4.9)	886 (5.4)
*Smoking history, no. (%)*			
Never smoked	4,335,259 (95.8)	4,320,036 (95.8)	15,223 (92.0)
Former smoker	51,171 (1.1)	50,912 (1.1)	259 (1.6)
Current smoker	138,773 (3.1)	137,699 (3.1)	1074 (6.5)
*Pack years of smoking, no. (%)*			
Never smoker	4,335,259 (95.8)	4,320,036 (95.8)	15,223 (92.0)
Former smoker & <10	40,881 (0.9)	40,729 (0.9)	152 (0.9)
Former smoker & ≥10	10,290 (0.2)	10,183 (0.2)	107 (0.7)
Current smoker & <10	91,170 (2.0)	90,685 (2.0)	485 (2.9)
Current smoker & ≥10	47,603 (1.1)	47,014 (1.0)	589 (3.6)
*Drinker*			
None	3,908,753 (86.4)	3,894,093 (86.4)	14,660 (88.6)
Mild	594,527 (13.1)	592,711 (13.2)	1816 (11.0)
Heavy	21,923 (0.5)	21,843 (0.48)	80 (0.5)
Regular physical activity	1,819,153 (40.2)	1,813,280 (40.2)	5873 (35.5)
*Body mass index, no. (%)*			
<18.5 kg/m^2^	102,774 (2.3)	102,319 (2.3)	455 (2.8)
18.5 to < 23 kg/m^2^	1,608,543 (35.6)	1,602,747 (35.6)	5796 (35.0)
23 to < 25 kg/m^2^	1,182,847 (26.1)	1,178,649 (26.1)	4198 (25.4)
25 to < 30 kg/m^2^	1,428,031 (31.6)	1,422,676 (31.6)	5355 (32.3)
≥30 kg/m^2^	203,008 (4.5)	202,256 (4.5)	752 (4.5)
*Co-morbid condition*			
Hypertension, no. (%)	1,915,482 (42.3)	1,906,972 (42.3)	8510 (51.4)
DM, no. (%)	622,758 (13.8)	619,999 (13.8)	2759 (16.7)
Dyslipidaemia, no. (%)	1,657,900 (36.6)	1,651,724 (36.6)	6176 (37.3)
SBP	125.3 ± 16	125.3 ± 16	127.4 ± 16.2
DBP	76.7 ± 10.0	76.7 ± 10.0	77.3 ± 10.1
Glucose	100.4 ± 23.9	100.4 ± 23.9	101.6 ± 24.7
Cholesterol	207.0 ± 38.8	207.0 ± 38.8	205.0 ± 39.1
*Income*			
Q1 (lowest)	1,277,758 (28.2)	1,273,270 (28.2)	4488 (27.1)
Q2	1,007,569 (22.3)	1,004,198 (22.3)	3371 (20.4)
Q3	1,056,997 (23.4)	1,053,003 (23.4)	3994 (24.1)
Q4 (highest)	1,182,879 (26.1)	1,178,176 (26.1)	4703 (28.4)

*SD* standard deviation.

Compared with the women not diagnosed with lung cancer, lung cancer cases had a higher mean age at study entry (65.9 vs. 61.3 years), a higher proportion of current and past smokers (6.5 and 1.6 vs. 3.1 and 1.1%), a tendency to exercise less (35.5 vs. 40.2%), more hypertension (51.4 vs. 42.3%) and more diabetes (16.7 vs. 13.8%). With respect to reproductive factors, only the duration of breastfeeding differed; the proportion of women breastfeeding for ≥1 year in lung cancer patients was 73.8%, which was higher than that among women not diagnosed with lung cancer (66.1%). The mean ages of menarche and menopause for lung cancer patients and the non-case group were 16.6 vs. 16.3 and 50.0 vs. 50.2 years, respectively, and the mean reproductive period was 33.4 vs. 33.9 years. Supplementary Table 1 provides a baseline characteristic analysis according to smoking status.

### Associations between reproductive factors and the risk of lung cancer

None of the menstrual or reproductive variables analysed showed a statistically significant association with lung cancer (Table 2). In multivariate-adjusted model 2, the HRs for lung cancer were 1.03 (95% CI, 0.98–1.09) for those whose age at menarche was ≥18 years compared with those aged ≤14 years, 1.02 (95% CI, 0.91–1.14) for those whose age at menopause was ≥55 years compared with those <40 years old, 0.95 (95% CI, 0.84–1.06) for the group having ≥2 parity compared with those with nulliparity, 0.95 (95% CI, 0.88–1.02) for the group breastfeeding for ≥1 year compared with those with no breastfeeding history, 1.04 (95% CI, 0.95–1.15) for the group on HRT for ≥5 years compared with the never-HRT group and 1.06 (95% CI, 0.99–1.13) for the patients who had used oral contraception for ≥1 year compared with never-users. Furthermore, when model 3 was adjusted for reproductive period instead of age at menarche and menopause as in model 2, similar trends were observed. The reproductive period was calculated as the interval between the age at menarche and the age at menopause, indicating a strong correlation among those variables. Therefore, we analysed them separately. Moreover, a sensitivity analysis restricted to non-smokers showed no statistically significant association between reproductive factors and the risk of lung cancer (Supplementary Table 2).

**Table 2:** Hazard ratios (HR) and 95% confidence intervals (CI) for the association between reproductive factors and the risk of lung cancer among postmenopausal women.

	Case no.	Duration (person-years)	IR per 100 000 person-years	HR (95% CI)		
				Model 1 (age-adjusted)	Model 2 (multivariable^a^)	Model 2 (multivariable^b^)
*Age at menarche*						
≤14 y	1925	2,958,968.8	65.1	1 (ref.)	1 (ref.)	
15–16 y	6173	2,718,665.8	78.7	1.02 (0.97–1.07)	1.02 (0.97–1.08)	
17 y	3365	7,840,631.0	87.4	0.98 (0.93–1.04)	0.99 (0.93–1.05)	
≥18 y	5093	9,111,375.6	96.8	1.03 (0.97–1.08)	1.03 (0.98–1.09)	
*Age at menopause*						
<40 y	354	332,191.7	106.6	1 (ref.)	1 (ref.)	
40–44 y	1084	1,077,353.8	100.6	0.98 (0.87–1.11)	0.99 (0.88–1.12)	
45–49 y	4266	5,248,111.7	81.3	0.93 (0.83–1.03)	0.95 (0.85–1.06)	
50–54 y	8745	10,955,113.3	79.8	0.93 (0.84–1.04)	0.96 (0.87–1.07)	
≥55 y	2107	2,298,204.9	91.7	0.98 (0.88–1.10)	1.02 (0.91–1.14)	
*Reproductive period*						
<30 y	2615	2,578,602.2	101.4	1 (ref.)		1 (ref.)
30–34 y	6775	7,951,785.0	85.2	0.97 (0.93–1.02)		0.98 (0.94–1.03)
35–39 y	5879	7,911,853.1	74.3	0.97 (0.93–1.02)		0.99 (0.95–1.04)
≥40 y	1287	1,468,735.2	87.6	1.02 (0.96–1.10)		1.05 (0.98–1.12)
*Parity*						
Nulliparous	377	466,390.6	80.8	1 (ref.)	1 (ref.)	1 (ref.)
1 child	970	1,410,842.3	68.8	0.95 (0.85–1.07)	1.00 (0.88–1.14)	1.00 (0.88–1.14)
≥2 children	15,209	18,033,742.6	84.3	0.84 (0.75–0.93)	0.95 (0.84–1.06)	0.95 (0.84–1.06)
*Duration of breastfeeding*						
Never	1062	1,520,411.3	69.9	1 (ref.)	1 (ref.)	1 (ref.)
<0.5 y	837	1,458,435.9	57.4	0.86 (0.79–0.94)	0.91 (0.82–1.00)	0.91 (0.82–1.00)
0.5 to <1 y	2439	3,478,988.5	70.1	0.88 (0.82–0.94)	0.95 (0.87–1.03)	0.95 (0.88–1.03)
≥1 y	12,218	13,453,139.8	90.8	0.87 (0.82–0.93)	0.95 (0.88–1.02)	0.95 (0.88–1.02)
*Hormone therapy*						
Never used	13,824	16,255,096.0	85.0	1 (ref.)	1 (ref.)	1 (ref.)
<2 y	1203	1,744,475.1	69.0	1.05 (0.99–1.12)	1.05 (0.99–1.11)	1.05 (0.99–1.11)
2 to <5 y	450	675,304.8	66.6	0.98 (0.89–1.07)	0.96 (0.88–1.06)	0.96 (0.88–1.06)
≥5 y	433	535,470.9	80.9	1.05 (0.96–1.16)	1.04 (0.95–1.15)	1.04 (0.95–1.15)
Missing	646	700,628.7	92.2	1.09 (1.01–1.18)	1.07 (0.98–1.16)	1.07 (0.98–1.16)
*Oral contraceptive use*						
Never used	13,282	16,005,619.4	83.0	1 (ref.)	1 (ref.)	1 (ref.)
<1 y	1345	176,6051.5	76.2	0.99 (0.94–1.05)	0.99 (0.94–1.05)	0.99 (0.94–1.05)
≥1 y	1043	1,171,489.9	89.0	1.07 (1.01–1.14)	1.06 (0.99–1.13)	1.06 (0.99–1.13)
Missing	886	967,814.6	91.6	1.07 (1.00–1.15)	1.05 (0.97–1.13)	1.05 (0.97–1.13)
*Pack years of smoking*						
Never smoker	15,223	19,118,652.5	79.6	1 (ref.)	1 (ref.)	1 (ref.)
Former smoker & <10	152	168,394.2	90.3	1.30 (1.11–1.52)	1.27 (1.08–1.49)	1.27 (1.08–1.49)
Former smoker & ≥10	107	44,052.2	242.9	2.65 (2.19–3.20)	2.59 (2.14–3.14)	2.59 (2.14–3.14)
Current smoker & <10	485	376,445.3	128.8	2.03 (1.85–2.22)	1.98 (1.80–2.17)	1.98 (1.80–2.17)
Current smoker & ≥10	589	203,431.2	289.5	3.48 (3.20–3.77)	3.40 (3.12–3.69)	3.40 (3.12–3.69)
*Alcohol consumption*						
None	14,660	17,293,429.6	84.8	1 (ref.)	1 (ref.)	1 (ref.)
Mild (<30 g/d)	1816	2,525,463.6	71.9	1.13 (1.08–1.19)	1.03 (0.98–1.09)	1.03 (0.98–1.09)
Heavy (≥30 g/d)	80	92,082.2	86.9	1.53 (1.23–1.91)	1.05 (0.84–1.31)	1.05 (0.84–1.31)
*Regular physical activity*						
No	10,683	11967393.3	89.3	1 (ref.)	1 (ref.)	1 (ref.)
Yes	5873	7943582.1	73.9	0.96 (0.93–0.99)	0.97 (0.94–1.00)	0.97 (0.94–1.00)
*Body mass index (kg/m*^*2*^*)*						
<18.5	455	427,226.4	106.5	1.07 (0.97–1.17)	1.02 (0.93–1.12)	1.02 (0.93–1.12)
18.5 to <23 kg/m^2^	5796	6,953,966.4	83.4	1 (ref.)	1 (ref.)	1 (ref.)
23 to <25 kg/m^2^	4198	5,253,687.4	79.9	0.94 (0.90–0.98)	0.95 (0.91–0.99)	0.95 (0.91–0.99)
25 to <30 kg/m^2^	5355	6,390,031.5	83.8	0.94 (0.90–0.97)	0.94 (0.91–0.98)	0.94 (0.91–0.98)
≥30	752	886,063.8	84.9	0.97 (0.90–1.04)	0.96 (0.88–1.03)	0.96 (0.88–1.03)
*Co-morbidity*						
*Hypertension*						
No	8046	11,397,844.2	70.6	1 (ref.)	1 (ref.)	1 (ref.)
Yes	8510	8,513,131.2	100.0	1.03 (1.00–1.07)	1.05 (1.01–1.08)	1.05 (1.01–1.08)
*Diabetes mellitus*						
No	13,797	17,214,825.2	80.2	1 (ref.)	1 (ref.)	1 (ref.)
Yes	2759	2,696,150.2	102.3	1.04 (1.00–1.09)	1.04 (0.99–1.08)	1.04 (0.99–1.08)
*Dyslipidaemia*						
No	10,380	12,762,248.2	81.3	1 (ref.)	1 (ref.)	1 (ref.)
Yes	6176	7,148,727.3	86.4	1.00 (0.97–1.03)	0.98 (0.95–1.02)	0.98 (0.95–1.02)
*Income*						
Q1 (lowest)	4488	5,481,769.8	81.9	1.04 (1.00–1.09)	1.00 (0.96–1.04)	1.00 (0.96–1.04)
Q2	3371	4,452,819.7	75.7	0.98 (0.94–1.03)	0.96 (0.92–1.00)	0.96 (0.92–1.00)
Q3	3994	4,749,847.9	84.1	0.98 (0.94–1.03)	0.97 (0.93–1.01)	0.97 (0.93–1.02)
Q4 (highest)	4703	5,226,537.9	90.0	1 (ref.)	1 (ref.)	1 (ref.)

*IR* incidence rate, *HR* hazard ratio, *CI* confidence interval.

^a^Adjusted for age, age at menarche and menopause, parity, duration of breastfeeding, duration of HRT, duration of oral contraceptive use, alcohol consumption, smoking, regular exercise, income, BMI, hypertension, diabetes mellitus, dyslipidaemia and cancer.

^b^Adjusted for age, reproductive period, parity, duration of breastfeeding, duration of HRT, duration of oral contraceptive use, alcohol consumption, smoking, regular exercise, income, BMI, hypertension, diabetes mellitus, dyslipidaemia and cancer.

### Other factors associated with lung cancer

Compared with never-smokers, current smokers were at a significantly increased risk for lung cancer (aHR 1.98; 95% CI, 1.80–2.17 for <10 pack years and aHR 3.40; 95% CI, 3.12–3.69 for ≥10 pack years). The risk of lung cancer in women who were former smokers also remained elevated compared with never-smokers (aHR 1.27; 95% CI, 1.08–1.49 for <10 pack years and aHR 2.59; 95% CI, 2.14–3.14 for ≥10 pack years). Hypertension was associated with a slightly elevated risk of lung cancer (aHR 1.05; 95% CI, 1.01–1.08). Alcohol consumption, physical activity, BMI, diabetes mellitus, dyslipidaemia and income level were not statistically significant in the multivariable analyses.

## Discussion

In the largest cohort study to date, we found that a wide range of reproductive history measures made no consistent contributions to lung cancer risk. The strengths of our study include an unprecedentedly large representative sample size and outcome incidence (more than 4.5 million women with 16,556 incident lung cancer cases), a comprehensive evaluation of the reproductive variables of interest and minimal follow-up losses (1.3%).

None of the menstrual variables analysed in our study showed a statistically significant association with lung cancer. Decades ago, a case–control study reported that a later age at menopause was associated with an increased lung cancer risk,^13,14^ whereas more recent studies found that a later age at menopause was associated with a reduced risk of lung cancer.^15,47^ With respect to menarche, Brinton et al.^15^ reported that early menarche was associated with an increased risk of adenocarcinoma, and a few other studies reported an increased risk for lung cancer with a later age at menarche, but the risk estimates were not statistically significant.^19,22^ Most other studies reported null associations.^16,20,22,23,26,48–54^ Weiss et al. reported that a longer reproductive period was associated with a decrease in the risk of lung cancer (HR 0.60 for 36 vs. <31 years; 95% CI, 0.39–0.93),^22^ but other studies reported null associations.^18,21,33^ Overall, previous studies showed that menstrual variables were not consistently associated with lung cancer risk, and our results support that finding.

Concerning parity, Kabat et al. reported an increased risk with increasing parity in Canada,^20^ but most other cohort studies reported an inverse association^19,21–23,55^ or null effect.^15,17,18,26,48,50,51,53,56,57^ A recent meta-analysis of 11 case–control and 5 cohort studies also found no overall relationship with parity.^58^ Our result of no association with parity concurs with that meta-analysis.

Only a few epidemiologic studies have examined the association between duration of breastfeeding and lung cancer, and their results showed null effects.^18,21,33^ Those studies were all conducted in East Asia, and our null result is consistent with theirs.

Previous findings about the association between HRT and lung cancer are conflicting, with studies reporting a reduced risk,^17,29,30^ an increased risk^27,28^ and a null effect.^15,16,19,23,25,31,32,49^ A systemic review that analysed 6 case–control studies showed a protective association between HRT and lung cancer,^59^ but a recent meta-analysis of 14 cohort studies found no statistically significant association between HRT and lung cancer.^60^ Our largest-ever cohort study confirms the null association.

The epidemiologic literature is also inconsistent with regard to the role of oral contraceptives in lung cancer risk. Some case–control studies^14,53,59^ noted that women who had used an oral contraceptive had a decreased risk relative to women who had not used one. But most other studies reported a null association.^18–20,22–26,51^ A systemic review^61^ that analysed nine case–control and five cohort studies showed no statistically significant association between oral contraceptive use and lung cancer, which is consistent with our findings.

While not the focus of our study, our data show that smoking is a definite risk factor for the development of lung cancer, producing a clear dose–response relationship. In East Asia, smoking prevalence in females has been relatively low due to a social atmosphere that deters women from smoking.^62^ Indeed, in our study, only 3.1 and 1.1% women identified themselves as current or past smokers. The other 95.8% were never-smokers at cohort entry, and 92.0% of lung cancer patients were never-smokers. However, the female smoking rate is increasing, especially in younger populations,^63^ and there are many hidden smokers.^62^ Some studies suggest that women are more susceptible to tobacco carcinogenesis than men.^64–66^ In the absence of other potent risk factors for women, our study’s results emphasise the importance of abstinence from smoking to prevent lung cancer in women.

In our study, having hypertension was slightly associated with an increase in lung cancer risk (aHR 1.05; 95% CI, 1.01–1.08) in women. Lindgren et al. reported that elevated blood pressure was associated with increased risk of lung cancer in smoking, hypertensive men,^67^ but most other cohort studies reported a null effect.^68–72^ The biological mechanism of such an association has rarely been investigated, but one recent study suggested that angiotensin-converting enzyme inhibitors are linked to a small increase in the risk of lung cancer and could be a potential confounder.^73^ Further studies are required to clarify this issue.

This study has several limitations. First, all the primary variables of interest were based on self-reported reproductive histories, and we therefore cannot exclude the possibility of bias caused by inaccurate recall. However, our data showed a null association, and it seems unlikely that recall bias could noticeably change that conclusion. Second, information on the components of HRT and oral contraceptives (e.g. oestrogen alone or oestrogen–progesterone) was unavailable. However, many studies, including the Women’s Health Initiative trials, reported that neither the use of oestrogen plus progestin nor oestrogen alone was associated with lung cancer incidence,^28,74,75^ and the HRT formula is unlikely to affect our conclusion of no association. Third, information about other possible risk factors for lung cancer (second-hand smoke exposure,^76–78^ indoor pollutants^79,80^ and a family history of lung cancers^14,81^) was also unavailable. Fourth, our study participants were limited to health-screening participants and might be healthier and more engaged in having a healthy lifestyle than the general population. Fifth, the follow-up period was relatively short at 4.4 years. Sixth, we could not include the pathological type of lung cancer because our data could not be linked to cancer registry data.

## Conclusions

Overall, this study found no statistically significant association between reproductive factors or hormone use, and the risk of lung cancer in postmenopausal Korean women, among whom smoking prevalence is very low. Further studies are warranted to discover additional risk factors for lung cancer in the female population.

## Supplementary information

Supplementary Table 1

Supplementary Table 2

## Data Availability

The data that support the findings of this study are available from NHIS. Restrictions apply to the availability of these data, which were used under license for this study. Data are available at https://nhiss.nhis.or.kr with the permission of NIHS.
