# Supplementary material for: Female reproductive factors and the risk of lung cancer in postmenopausal women: a nationwide cohort study
Source: Br J Cancer. 2020 Mar 17;122(9):1417–24. doi: 10.1038/s41416-020-0789-7 (PMC7188895; doi:10.1038/s41416-020-0789-7)
Supplement: Supplementary file 1 — Supplementary Table 1 [file 41416_2020_789_MOESM1_ESM.docx]

Supplementary Table 1. Baseline characteristics analysis according to smoking status

| Variable | | Never smoker  (N = 4,335,259) | | | Former smoker  (N = 51,171) | | Current smoker  (N = 138,773) | |
| --- | --- | --- | --- | --- | --- | --- | --- | --- |
| Mean age, y (SD) | 61.4 (8.7) | | 59.8 (9.1) | | | 58.9 (8.7) | | |
| Age at menarche, No. (%) |  | |  | | |  | | |
| Mean (SD) | 16.3 (1.9) | | | 16.0 (1.9) | | | | 16.2 (1.9) |
| ≤ 14 y | 676,811 (15.6) | | | 11,187 (21.9) | | | | 24,181 (17.4) |
| 15-16 y | 1,732,257 (40.0) | | | 20,635(40.3) | | | | 53,974 (38.9) |
| 17 y | 820,834 (18.9) | | | 8,479(16.6) | | | | 24,810 (17.9) |
| ≥ 18 y | 1,105,357 (25.5) | | | 10,870(21.2) | | | | 35,808 (25.8) |
| Age at menopause, No. (%) |  |  |  |  |  |  |  |  |
| Mean (SD) | 50.3 (4.0) | | | 49.9 (4.2) | | | | 49.6 (4.2) |
| < 40 y | 69,138 (1.6) | | | 1,057 (2.1) | | | | 3,279 (2.4) |
| 40-44 y | 224,370 (5.2) | | | 3,250 (6.4) | | | | 9,964 (7.2) |
| 45-49 y | 1,115,211 (25.7) | | | 14,403 (28.2) | | | | 42,570 (30.7) |
| 50-54 y | 2,412,929 (55.7) | | | 27,150 (53.1) | | | | 70,654 (50.9) |
| ≥ 55 y | 513,611 (11.9) | | | 5,311 (10.4) | | | | 12,306 (8.9) |
| Reproductive period, No. (%) |  | | |  | | | |  |
| Mean (SD) | 34.0 (4.4) | | | 34.0 (4.5) | | | | 33.3 (4.6) |
| < 30 | 534,119 (12.3) | | | 6,677 (13.1) | | | | 22,011 (15.9) |
| 30-34 y | 1,691,407 (39.0) | | | 19,405 (37.9) | | | | 57,212 (41.2) |
| 35-39 y | 1,772,240 (40.9) | | | 20,974 (41.0) | | | | 51,056 (36.8) |
| ≥ 40 | 337,493 (7.78) | | | 4,115 (8.0) | | | | 8,494 (6.1) |
| Parity, No. (%) |  |  |  |  |  |  |  |  |
| Nulliparous | 312,655 (7.2) | | | 9,610 (18.8) | | | | 25,196 (18.2) |
| 1 child | 3,934,771 (90.8) | | | 38,206 (74.7) | | | | 105,541 (76.1) |
| ≥ 2 children | 87,833 (2.0) | | | 3,355 (6.6) | | | | 8,036 (5.8) |
| Duration of breastfeeding,  No. (%) |  |  |  |  |  |  |  |  |
| Never | 341,810 (7.9) | | | 6,472 (12.7) | | | | 14,293 (10.3) |
| < 0.5 y | 772,500 (17.8) | | | 9,629 (18.8) | | | | 22,536 (16.2) |
| 0.5 to < 1 y | 2,886,741 (66.6) | | | 26,123 (51.1) | | | | 78,861 (56.8) |
| ≥ 1 y | 334,208 (7.7) | | | 8,947 (17.5) | | | | 23,083 (16.6) |
| Hormone therapy, No. (%) |  |  |  |  |  |  |  |  |
| Never used | 3,548,968 (81.9) | | | 37528(73.34) | | | | 109528(78.93) |
| < 2 y | 371,945 (8.6) | | | 6285(12.28) | | | | 14252(10.27) |
| 2 to < 5 y | 142,736 (3.3) | | | 2560(5) | | | | 5417(3.9) |
| ≥ 5 y | 113,540 (2.6) | | | 2189(4.28) | | | | 4161(3) |
| Missing | 158,070 (3.7) | | | 2609(5.1) | | | | 5415(3.9) |
| Oral contraceptive use,  No. (%) |  |  |  |  |  |  |  |  |
| Never used | 3,505,336 (80.9) | | | 36,347 (71.0) | | | | 105,150 (75.8) |
| < 1 y | 375,901 (8.7) | | | 6,316 (12.3) | | | | 14,894 (10.7) |
| ≥ 1 y | 244,250 (5.6) | | | 5,309 (10.4) | | | | 11,720 (8.5) |
| Missing | 209,772 (4.8) | | | 3,199 (6.3) | | | | 7,009 (5.1) |
| Pack years of smoking,  No. (%) |  |  |  |  |  |  |  |  |
| Never smoker | 4,335,259 (100) | | |  | | | |  |
| Former smoker & < 10 |  | | | 40,881 (79.9) | | | |  |
| Former smoker & ≥ 10 |  | | | 10,290 (20.1) | | | |  |
| Current smoker & < 10 |  | | |  | | | | 91,170 (65.7) |
| Current smoker & ≥ 10 |  | | |  | | | | 47,603 (34.3) |
| Drinker |  |  |  |  |  |  |  |  |
| Non | 3,795,294 (87.5) | | | 32,269 (63.1) | | | | 81,190 (58.5) |
| Mild (< 30 g/d) | 525,804 (12.1) | | | 17,580 (34.4) | | | | 51,143 (36.9) |
| Heavy (≥ 30 g/d) | 14,161 (0.3) | | | 1,322 (2.6) | | | | 6,440 (4.6) |
| Regular physical activity |  | | |  | | | |  |
| No | 2,586,893 (59.7) | | | 27,766 (54.3) | | | | 91,391 (65.9) |
| Yes | 1,748,366 (40.3) | | | 23,405 (45.7) | | | | 47,382 (34.1) |
| Body mass index, No (%) |  |  |  |  |  |  |  |  |
| < 18.5 kg/m^2^ | 94,385 (2.2) | | | 1,513 (3.0) | | | | 6,876 (5.0) |
| 18.5 to < 23 kg/m^2^ | 1,531,054 (35.3) | | | 19,351 (37.8) | | | | 58,138 (41.9) |
| 23 to < 25 kg/m^2^ | 1,139,703 (26.3) | | | 12,270 (24.0) | | | | 30,874 (22.3) |
| 25 to < 30 kg/m^2^ | 1,376,230 (31.8) | | | 15,233 (29.8) | | | | 36,568 (26.4) |
| ≥ 30 kg/m^2^ | 193,887 (4.5) | | | 2,804 (5.5) | | | | 6,317 (4.6) |
| Co-morbid condition |  | | |  | | | |  |
| Hypertension, No (%) | 1,844,629 (42.6) | | | 20,241 (39.6) | | | | 50,612 (36.5) |
| DM, No (%) | 593,409 (13.7) | | | 7,971 (15.6) | | | | 21,378 (15.4) |
| Dyslipidemia, No (%) | 1,581,766 (36.5) | | | 21,240 (41.5) | | | | 54,894 (39.6) |
| SBP | 125.4 ± 16.0 | | | 122.6 ± 16.2 | | | | 121.4 ± 16.1 |
| DBP | 76.7 ± 10.0 | | | 75.4 ± 10.3 | | | | 75.2 ± 10.3 |
| Glucose | 100.3 ± 23.7 | | | 101.4 ± 25.4 | | | | 102.0 ± 27.3 |
| Cholesterol | 206.8 ± 38.7 | | | 209.2 ± 40.6 | | | | 211.4 ± 41.2 |
| Income |  | | |  | | | |  |
| Q1(lowest) | 1,200,305 (27.7) | | | 19,007 (37.1) | | | | 58,446 (42.1) |
| Q2 | 961,718 (22.2) | | | 11,677 (22.8) | | | | 34,174 (24.6) |
| Q3 | 1,019,788 (23.5) | | | 10,544 (20.6) | | | | 26,665 (19.2) |
| Q4(highest) | 1,153,448 (26.6) | | | 9,943 (19.4) | | | | 19,488 (14.0) |

SD, standard deviation
