# Supplementary material for: Female reproductive factors and the risk of lung cancer in postmenopausal women: a nationwide cohort study
Source: Br J Cancer. 2020 Mar 17;122(9):1417–24. doi: 10.1038/s41416-020-0789-7 (PMC7188895; doi:10.1038/s41416-020-0789-7)
Supplement: Supplementary file 2 — Supplementary Table 2 [file 41416_2020_789_MOESM2_ESM.docx]

Supplementary Table 2. Sensitivity analysis for the association between reproductive factors and the risk of lung cancer among non-smokers

|  | Case No. | | Duration  (person-years) | IR per 100 000 person-years | HR (95% CI) | | |
| --- | --- | --- | --- | --- | --- | --- | --- |
|  |  |  |  |  | Model 1  (Age-adjusted) | Model 2  (multivariable†) | Model 2  (multivariable‡) |
| Age at menarche |  | |  |  |  |  |  |
| ≤ 14 y | 1,770 | | 2,817,148.5 | 62.8 | 1 (ref.) | 1 (ref.) |  |
| 15-16 y | 5,657 | | 7,533,258.6 | 75.1 | 1.01(0.96-1.07) | 1.01 (0.96-1.07) |  |
| 17 y | 3,108 | | 3,707,176.1 | 83.8 | 0.99 (0.93-1.05) | 0.99 (0.93-1.05) |  |
| ≥ 18 y | 4,688 | | 5,061,069.3 | 92.6 | 1.03(0.98-1.09) | 1.03 (0.98-1.09) |  |
| Age at menopause |  | |  |  |  |  |  |
| < 40 y | 306 | | 313,581.6 | 97.6 | 1 (re f.) | 1 (ref.) |  |
| 40-44 y | 948 | | 1,021,421.7 | 92.8 | 0.99 (0.867-1.12) | 0.99 (0.87-1.13) |  |
| 45-49 y | 3,870 | | 5,007,328.4 | 77.3 | 0.96 (0.85-1.07) | 0.96 (0.85-1.08) |  |
| 50-54 y | 8,126 | | 10,550,290.5 | 77.0 | 0.98 (0.87-1.10) | 0.98 (0.88-1.103) |  |
| ≥ 55 y | 1,973 | | 2,226,030.3 | 88.6 | 1.04 (0.92-1.17) | 1.04 (0.93-1.18) |  |
| Reproductive period |  | |  |  |  |  |  |
| < 30 y | 2,326 | | 2,455,159.3 | 94.7 | 1 (ref.) |  | 1 (ref.) |
| 30-34 y | 6,230 | | 7,627,121.6 | 81.7 | 0.99 (0.95-1.04) |  | 0.99 (0.95-1.04) |
| 35-39 y | 5,468 | | 7,618,135.0 | 71.8 | 1 (0.95-1.05) |  | 1.00 (0.95-1.05) |
| ≥ 40 y | 1,199 | | 1,418,236.6 | 84.5 | 1.06 (0.99-1.13) |  | 1.06 (0.99-1.14) |
| Parity |  | |  |  |  |  |  |
| Nulliparous | 313 | | 414,928.5 | 75.4 | 1 (ref.) | 1 (ref.) | 1 (ref.) |
| 1 child | 833 | | 1,273,912.1 | 65.4 | 0.98 (0.86-1.11) | 1.01 (0.88-1.16) | 1.01 (0.88-1.16) |
| ≥ 2 children | 14,077 | | 17,429,812.0 | 80.8 | 0.879 (0.79-0.98) | 0.91 (0.81-1.04) | 0.91 (0.81-1.03) |
| Duration of breastfeeding | | |  |  |  |  |  |
| Never | | 904 | 1,390,208.5 | 65.0 | 1 (ref.) | 1 (ref.) | 1 (ref.) |
| < 0.5 y | | 749 | 1,378,104.6 | 54.4 | 0.88 (0.80-0.97) | 0.9 (0.81-1.00) | 0.90 (0.81-1.00) |
| 0.5 to < 1 y | | 2,271 | 3,346,736.2 | 67.9 | 0.92 (0.85-1.00) | 0.96 (0.88-1.04) | 0.96 (0.88-1.04) |
| ≥ 1 y | | 11,299 | 13,003,603.2 | 86.9 | 0.92 (0.85-0.98) | 0.96 (0.88-1.03) | 0.96 (0.89-1.04) |
| Hormone therapy | |  |  |  |  |  |  |
| Never used | | 12,731 | 15,642,422.8 | 81.4 | 1 (ref.) | 1 (ref.) | 1 (ref.) |
| < 2 y | | 1,116 | 1,658,151.0 | 67.3 | 1.06 (0.99-1.13) | 1.06 (0.99-1.12) | 1.05 (0.99-1.12) |
| 2 to < 5 y | | 403 | 641,185.6 | 62.9 | 0.95 (0.86-1.05) | 0.94 (0.85-1.04) | 0.94 (0.85-1.04) |
| ≥ 5 y | | 395 | 508,295.7 | 77.7 | 1.05 (0.95-1.16) | 1.04 (0.94-1.15) | 1.04 (0.94-1.15) |
| Missing | | 578 | 668,597.5 | 86.5 | 1.07 (0.99-1.17) | 1.05 (0.96-1.15) | 1.05 (0.96-1.15) |
| Oral contraceptive use | |  |  |  |  |  |  |
| Never used | | 12,239 | 15,413,049.9 | 79.4 | 1 (ref.) | 1 (ref.) | 1 (ref.) |
| < 1 y | | 1,238 | 1,678,909.7 | 73.7 | 1.00 (0.94-1.06) | 0.99 (0.94-1.05) | 0.99 (0.94-1.05) |
| ≥ 1 y | | 941 | 1,101,305.3 | 85.4 | 1.06 (0.99-1.13) | 1.06 (0.99-1.13) | 1.06 (0.99-1.13) |
| Missing | | 805 | 925,387.6 | 87.0 | 1.06 (0.99-1.14) | 1.05 (0.97-1.13) | 1.05 (0.97-1.13) |
| Alcohol consumption | |  |  |  |  |  |  |
| Non | | 13,727 | 16,815,468.6 | 81.6 | 1 (ref.) | 1 (ref.) | 1 (ref.) |
| Mild (< 30 g/d) | | 1,453 | 2,242,728.7 | 64.8 | 1.04 (0.98-1.10) | 1.04 (0.98-1.10) | 1.04 (0.98-1.10) |
| Heavy (≥ 30 g/d) | | 43 | 60,455.2 | 71.1 | 1.22 (0.91-1.65) | 1.21 (0.90-1.64) | 1.21 (0.90-1.64) |
| Regular physical activity | | |  |  |  |  |  |
| No | | 9,743 | 11,471,976.1 | 84.9 | 1 (ref.) | 1 (ref.) | 1 (ref.) |
| Yes | | 5,480 | 7,646,676.4 | 71.7 | 0.98 (0.94-1.01) | 0.97 (0.94-1.01) | 0.97 (0.94-1.01) |
| Body mass index (kg/m^2^) | | |  |  |  |  |  |
| < 18.5 | | 343 | 393,745.1 | 87.1 | 0.92 (0.83-1.03) | 0.93 (0.83-1.03) | 0.93 (0.83-1.03) |
| 18.5 to < 23 kg/m^2^ | | 5,249 | 6,632,947.5 | 79.1 | 1 (ref.) | 1 (ref.) | 1 (ref.) |
| 23 to < 25 kg/m^2^ | | 3,919 | 5,071,759.5 | 77.3 | 0.96 (0.92-1.00) | 0.95 (0.91-0.99) | 0.95 (0.91-0.99) |
| 25 to < 30 kg/m^2^ | | 5,009 | 6,171,371.4 | 81.2 | 0.96 (0.92-1.00) | 0.95 (0.91-0.99) | 0.95 (0.91-0.99) |
| ≥ 30 | | 703 | 848,829.0 | 82.8 | 0.99 (0.92-1.07) | 0.97 (0.90-1.05) | 0.97 (0.90-1.05) |
| Co morbidity | |  |  |  |  |  |  |
| Hypertension | |  |  |  |  |  |  |
| No | | 7,366 | 10,902,090.8 | 67.6 | 1 (ref.) | 1 (ref.) | 1 (ref.) |
| Yes | | 7,857 | 8,216,561.7 | 95.6 | 1.04 (1.01-1.08) | 1.05(1.01-1.08) | 1.05 (1.01-1.08) |
| Diabetes Mellitus | |  |  |  |  |  |  |
| No | | 12,737 | 16,541,562.7 | 77.0 | 1 (ref.) | 1 (ref.) | 1 (ref.) |
| Yes | | 2,486 | 2,577,089.8 | 96.5 | 1.03 (0.98-1.07) | 1.03 (0.98-1.07) | 1.03 (0.98-1.07) |
| Dyslipidemia | |  |  |  |  |  |  |
| No | | 9,598 | 12,282,163.4 | 78.1 | 1 (ref.) | 1 (ref.) | 1 (ref.) |
| Yes | | 5,625 | 6,836,489.1 | 82.3 | 0.99 (0.96-1.02) | 0.98 (0.95-1.01) | 0.98 (0.95-1.01) |
| Income | |  |  |  |  |  |  |
| Q1(lowest) | | 3,981 | 5,169,870.2 | 77.0 | 1.00 (0.96-1.05) | 1.00 (0.96-1.04) | 1.00 (0.96-1.04) |
| Q2 | | 3,087 | 4,258,759.2 | 72.5 | 0.96 (0.92-1.01) | 0.96 (0.92-1.00) | 0.96 (0.92-1.01) |
| Q3 | | 3,708 | 4,590,739.6 | 80.8 | 0.97 (0.93-1.01) | 0.97 (0.93-1.01) | 0.97 (0.93-1.01) |
| Q4(highest) | | 4,447 | 5,099,283.5 | 87.2 | 1 (ref.) | 1 (ref.) | 1 (ref.) |

IR, incidence rate; HR, hazard ratio; CI, confidence interval.

† Adjusted for age, age at menarche and menopause, parity, duration of breastfeeding, duration of HRT, duration of oral contraceptive use, alcohol consumption, smoking, regular exercise, income, BMI, hypertension, diabetes mellitus, dyslipidemia, and cancer.

‡ Adjusted for age, reproductive period, parity, duration of breastfeeding, duration of HRT, duration of oral contraceptive use, alcohol consumption, smoking, regular exercise, income, BMI, hypertension, diabetes mellitus, dyslipidemia, and cancer
